# Supplementary material for: Dynamic clustering of genomics cohorts beyond race, ethnicity—and ancestry
Source: BMC Med Genomics. 2025 May 15;18:87. doi: 10.1186/s12920-025-02154-z (PMC12082885; doi:10.1186/s12920-025-02154-z)
Supplement: Supplementary file 2 — Supplementary Material 2. [file 12920_2025_2154_MOESM2_ESM.pdf]

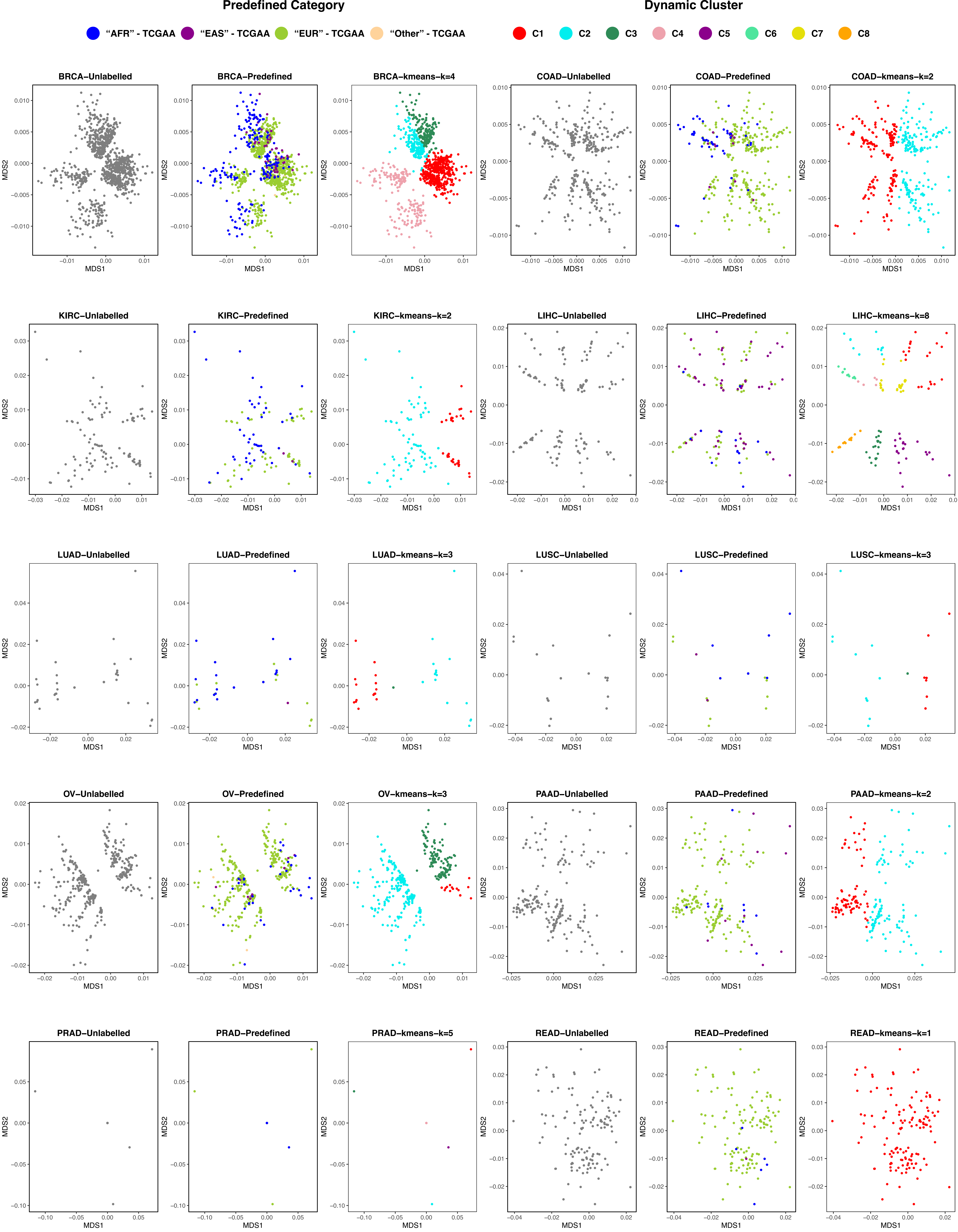

Supplementary Figure 2. COSMIC-based dynamic clustering across cancer types. Colors correspond to: no labels, predefined categories, and K-means clustering with "optimal" k.
