# Supplementary material for: Dynamic clustering of genomics cohorts beyond race, ethnicity—and ancestry
Source: BMC Med Genomics. 2025 May 15;18:87. doi: 10.1186/s12920-025-02154-z (PMC12082885; doi:10.1186/s12920-025-02154-z)
Supplement: Supplementary file 9 — Supplementary Material 9. [file 12920_2025_2154_MOESM9_ESM.pdf]

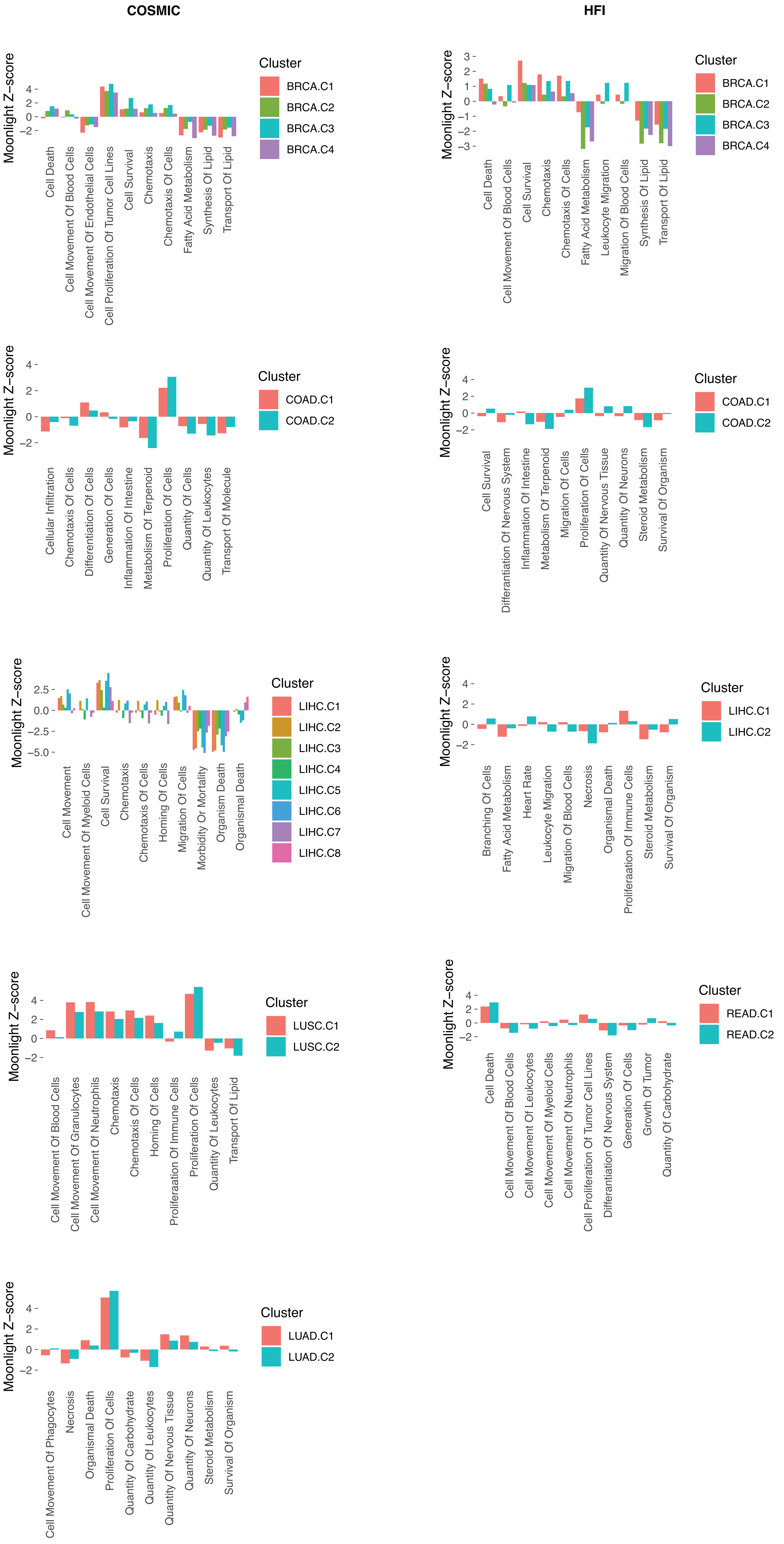

Supplementary Figure 9. Top Moonlight results of differentially expressed gene programs across cancer types and settings.
