# Supplementary material for: Dynamic clustering of genomics cohorts beyond race, ethnicity—and ancestry
Source: BMC Med Genomics. 2025 May 15;18:87. doi: 10.1186/s12920-025-02154-z (PMC12082885; doi:10.1186/s12920-025-02154-z)
Supplement: Supplementary file 10 — Supplementary Material 10. [file 12920_2025_2154_MOESM10_ESM.pdf]

LIHC-HFI-C1

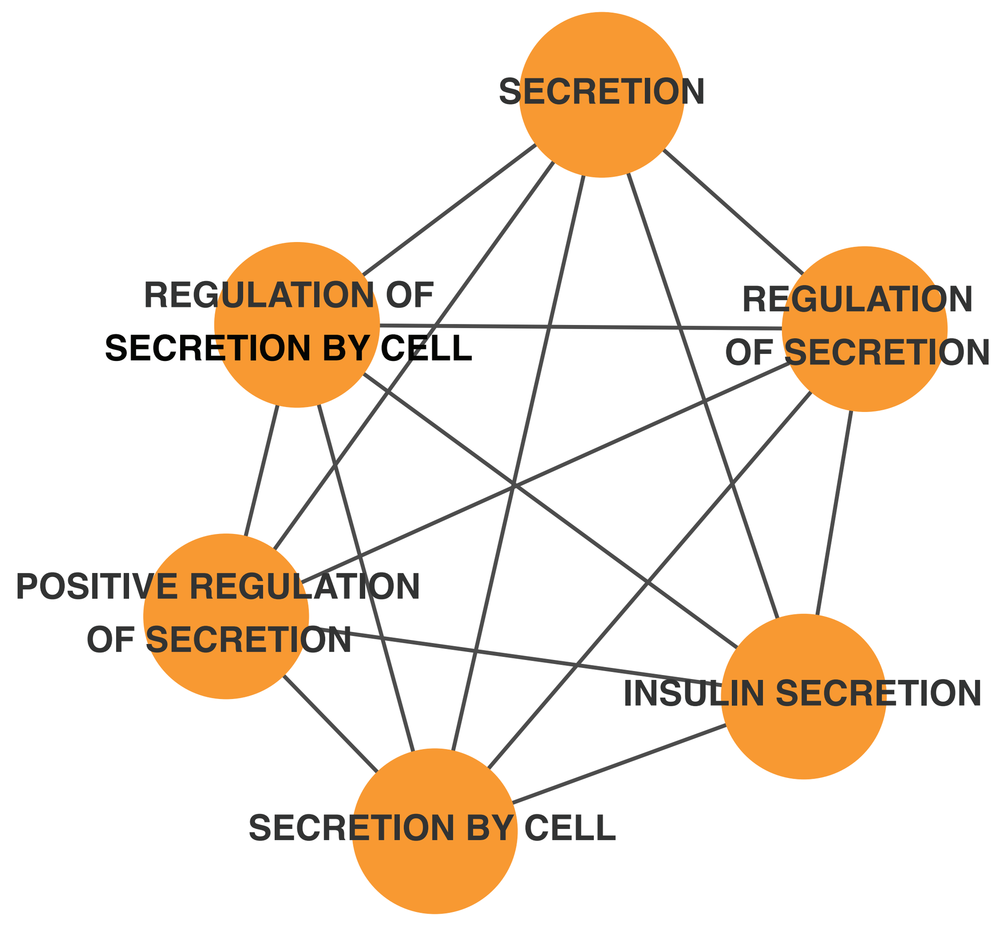

LUSC-COSMIC-C2

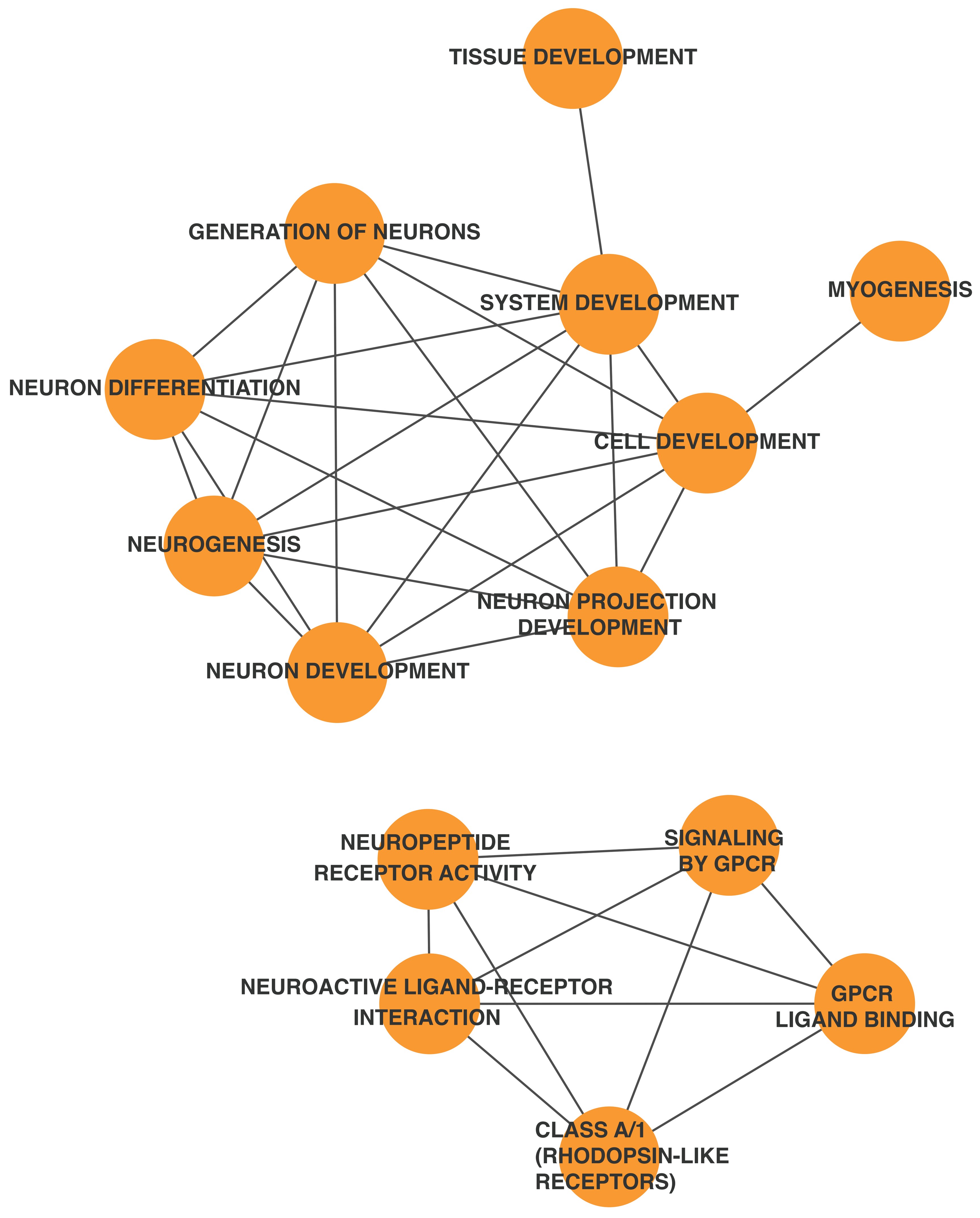

READ-HFI-C2

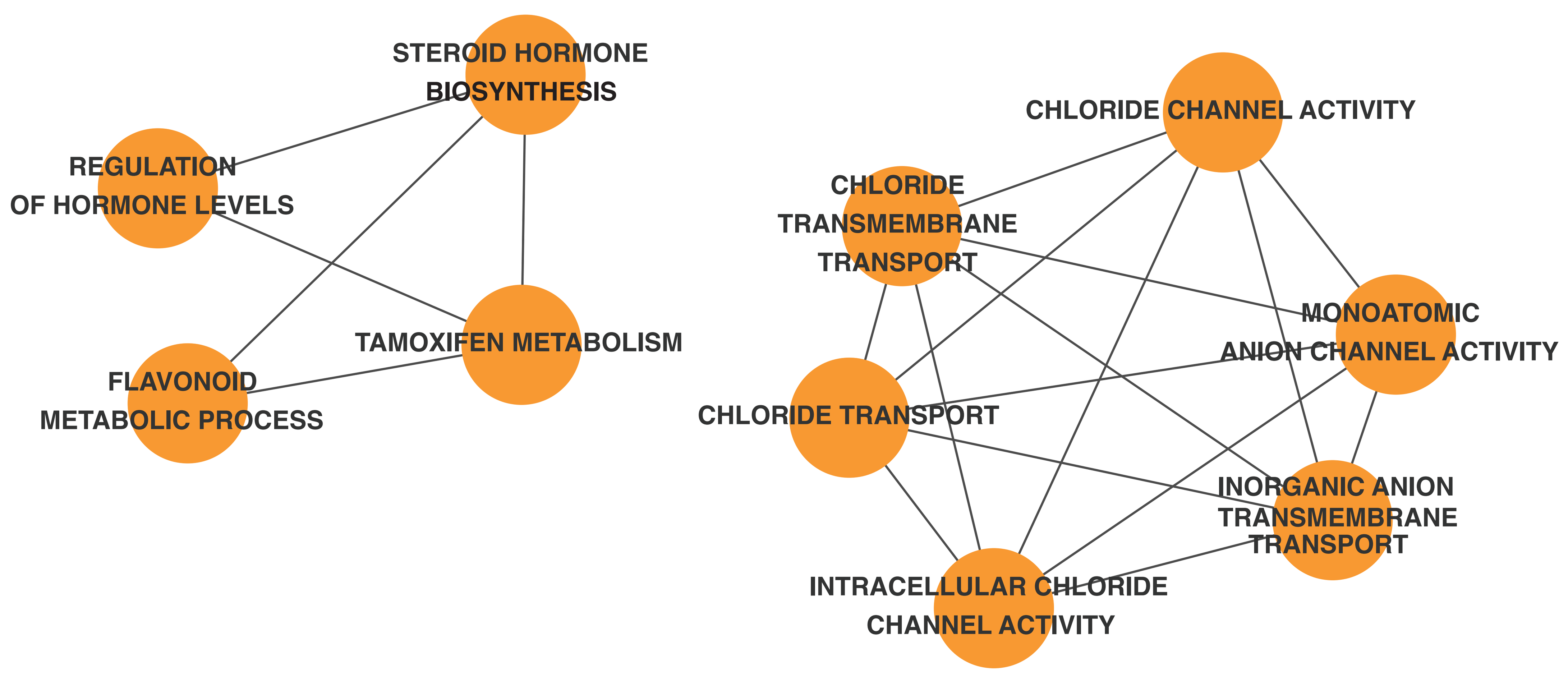

Supplementary Figure 10. Non-cancer signifiers significantly associated with single dynamic clusters in LIHC-HFI-C1, LUSC-COSMIC-C2, and READ-HFI-C2
